# Schlafen14 Impairs HIV-1 Expression in a Codon Usage-Dependent Manner

**DOI:** 10.3390/v16040502

**Published:** 2024-03-25

**Authors:** Carlos Valenzuela, Sergio Saucedo, Manuel Llano

**Affiliations:** 1 Biological Sciences Department, The University of Texas at El Paso, El Paso, TX 79968, USA; carlos-ruizvalenzuela@ouhsc.edu; 2 Paul L. Foster School of Medicine, Texas Tech University Health Sciences Center, El Paso, TX 79905, USA; sergsauc@ttuhsc.edu

**Keywords:** HIV-1, Schlafen14, translation, codon usage bias, ribosome

## Abstract

Schlafen (SLFN) is a family of proteins upregulated by type I interferons with a regulatory role in translation. Intriguingly, SLFN14 associates with the ribosome and can degrade rRNA, tRNA, and mRNA in vitro, but a role in translation is still unknown. Ribosomes are important regulatory hubs during translation elongation of mRNAs rich in rare codons. Therefore, we evaluated the potential role of SLFN14 in the expression of mRNAs enriched in rare codons, using HIV-1 genes as a model. We found that, in a variety of cell types, including primary immune cells, SLFN14 regulates the expression of HIV-1 and non-viral genes based on their codon adaptation index, a measurement of the synonymous codon usage bias; consequently, SLFN14 inhibits the replication of HIV-1. The potent inhibitory effect of SLFN14 on the expression of the rare codon-rich transcript HIV-1 Gag was minimized by codon optimization. Mechanistically, we found that the endoribonuclease activity of SLFN14 is required, and that ribosomal RNA degradation is involved. Therefore, we propose that SLFN14 impairs the expression of HIV-1 transcripts rich in rare codons, in a catalytic-dependent manner.

## 1. Introduction

Codon usage is one of the factors influencing the amount of polypeptide produced per mRNA in bacteria and eukaryotic cells, and tRNA abundance seems to be a major factor in this process [1,2,3,4,5,6,7,8,9,10,11,12]. The relative deficiency of charged tRNAs, associated with the translation of transcripts rich in rare codons, causes ribosome pausing, reducing the translation elongation rate and leading to ribosome stalling [7,13,14,15,16]. This event decreases global protein synthesis by premature termination and degradation of the truncated protein through the ribosome-associated protein quality control [12,17,18], degradation of the associated mRNAs through the No-go decay mechanism [19,20,21], rRNA degradation through the nonfunctional 18S rRNA decay pathway, or by inhibiting translation initiation via the integrated stress response [22].

The tRNA repertoire is modified during stress [23,24,25,26,27,28,29,30], including viral infection [31,32,33,34,35]. The innate immune system has evolved mechanisms to regulate protein expression based on rare codon usage bias through the effect of the type I interferon (IFN)-induced protein SLFN11. By tRNA degradation, SLFN11 negatively regulates expression of proteins encoded by transcripts with bias towards rare codons [31,32,36,37]. SLFN13 [38] and SLFN12 [39] also degrade tRNAs, whereas SLFN5 and SLFN2 bind to tRNA without degrading them, and through this interaction, SLFN2 protects against stress-induced, angiogenin-mediated tRNA degradation [27,40].

Another member of the SLFN family, SLFN14, was discovered as a stalled ribosome-associated endoribonuclease, and the purified protein was shown to degrade ribosomes, tRNAs, and mRNA in vitro [41,42,43]. Furthermore, SLFN14 was found to degrade LINE-1 mRNA [44] in cells. SLFN14 was also reported to impair the expression of two varicella zoster virus proteins, immediate-early 62, glycoprotein E [44,45], and the nucleoprotein from the influenza virus [45]. Intriguingly, all these viral proteins are encoded by transcripts enriched in rare codons that exhibit low codon adaptation indexes (CAI), a measurement of the synonymous codon usage bias [46]. For example, varicella zoster virus proteins, immediate-early 62 and glycoprotein E have a CAI of 0.72 and 0.7, respectively, and the nucleoprotein from the influenza virus has a CAI of 0.753. Therefore, we postulated that SLFN14 could inhibit translation of transcripts with bias toward rare codons in response to constraints that these transcripts likely encounter during translation elongation, i.e., ribosome stalling. To evaluate our hypothesis, we selected as a model the HIV-1 genome, which is rich in adenine nucleotides determining a codon usage bias that importantly differs from that of the host [47,48,49].

Our data indicate for the first time that SLFN14 potently inhibits expression of transcripts rich in rare codons, i.e., HIV-1 Gag and firefly luciferase; but not the expression of those poor in rare codons, i.e., codon-optimized HIV-1 Gag, HIV-1 Tat, cyan, monomeric (m) cherry, and green fluorescent proteins, and human CD4. As a consequence, HIV-1 replication is inhibited by SLFN14. The anti-HIV-1 effect was observed in primary monocytes and CD4+ T lymphocyte, and in cancer (SUP-T1) and transformed (HEK293T) cell lines. This activity is type I IFN-independent, but requires the endoribonuclease function of SLFN14. Expression of SLFN14 was associated with ribosome degradation in cells co-expressing wild-type but not codon-optimized HIV-1 Gag mRNA. In sum, our results indicate a novel function of SLFN14 in restricting expression of transcripts rich in rare codons.

## 2. Materials and Methods

**Cell lines.** SUP-T1, CEM, and MOLT-3 cells and primary immune cells were grown in RPMI 1640 medium supplemented with 10% heat-inactivated fetal calf serum, 2 mM L-glutamine, and 1% penicillin-streptomycin, while HEK293T cells were grown in Dulbecco’s modified Eagle’s medium (DMEM) supplemented with 10% heat-inactivated fetal calf serum, 2 mM L-glutamine, and 1% penicillin-streptomycin. All the cell lines used were previously obtained from ATCC.

**Expression plasmids.** pHluc was derived from pNL4-3luc-R-E- as described in [50]. This single-round infection HIV-1 expresses LTR-driven firefly luciferase from the *nef* slot, lacks VPR expression, and has a 426 nt deletion in the *env* gene. FLAG-tagged human and mouse SLFN14 (Origene, RC226257 and MR225976) were expressed from pCMV6-Entry. Empty plasmid was derived from the human SLFN14 expression plasmid by deleting the entire open reading frame (2.8 kb) by digestion Sal I/Xho I and religation of the backbone (4.8 kbs). FLAG-tagged human SLFN14 D249A was generated by site-directed mutagenesis with the QuickChange Lightning Site-Directed Mutagenesis Kit (Agilent, Santa Clara, CA, USA) using reverse primer SS1 (5′-atccaccccaatgaggacatatcc-3′) and forward primer SS2 (5′-gCtaagagcaaagaagtggttggatg-3′); the point mutation is indicated in upper case in the primer sequence. The entire sequence of the SLFN14 D249A cDNA was verified by overlapping DNA sequencing. Wild-type Gag was expressed from pCMVΔR8.91 (a gift of D. Trono) and codon-optimized Gag from pARP-8675 (NIH AIDS Reagent Program). This construct expresses a codon-optimized Gag pre-protein from HIV-1 clone 96ZM651.8 [51]. The cyan fluorescent protein (CFP) expression plasmid was pECFP-C1 (Clontech, Mountain View, CA, USA). Plasmids pCAGGS-CD4-Myc (Addgene, Watertown, MA, USA, 58537), and pRP-mCherry/Puro-CAG > hCXCR4 (VectorBuilder, Chicago, IL, USA, VB900125-2200scz) were used to express CD4 and CXCR4, respectively. The CXCR4 expression plasmid contains an independent mCherry expression cassette (bicistronic plasmid). pCI Luc contains firefly luciferase cDNA cloned MluI/Xba I in pCI (Promega, Madison, WI, USA). pNLENG1-ES-IRES (a gift of D.N. Levy, NYU) encodes a single-round infection HIV-1 (HIVeGFP) that contains two stop codons in *env*, and the eGFP open reading frame is inserted between the *env* and *nef* sequences. [52]. pNL4-3 encodes a wild-type HIV-1 (strain NY5/BRU, LAV-1).

**Generation of viruses.** Procedures previously described [53,54] were used for production of NL4-3 HIV-1 virus. Briefly, HEK293T cells were co-transfected by calcium phosphate with 15 ug of pNL4-3. Seventy-two hours after transfection, the viral supernatant was harvested.

**Immunoblotting.** HEK293T cells (3 × 10^6^) were lysed in 100 μL of Laemmli sample buffer (12 mM Tris-Cl, pH 6.8, 0.4% SDS, 2% glycerol, 1% β-mercaptoethanol, 0.002% bromophenol blue). SUP-T1, CEM and MOLT-3 cells, peripheral blood mononuclear cells (PBMCs), CD4+ T lymphocytes, and monocytes were lysed for 15 min on ice in 100 μL of CSK I buffer [55] (10 mM PIPES [piperazine-*N*,*N*′-bis(2-ethanesulfonic acid)] (pH 6.8), 100 mM NaCl, 1 mM EDTA, 300 mM sucrose, 1 mM MgCl_2_, 1 mM dithiothreitol (DTT), 0.5% Triton X-100) containing protease inhibitors (final concentrations of 2 μg/mL leupeptin, 5 μg/mL aprotinin, 1 mM phenylmethylsulfonyl fluoride, and 1 μg/mL pepstatin A). Cell lysates were centrifuged at 22,000× *g* for 3 min at 4 °C, and the supernatant mixed with Laemmli sample buffer, boiled for 10 min, and saved at −80 °C for further analysis. Cell lysates (15 μL) were resolved by SDS-PAGE and transferred overnight to polyvinylidene difluoride (PVDF) membranes at 100 mA at 4 °C. Membranes were blocked with Tris-buffered saline (TBS) containing 10% milk for 1 h and then incubated with the corresponding primary antibody diluted in TBS-5% milk-0.05% Tween 20 (antibody dilution buffer). FLAG-tagged mouse and human SLFN14 was detected with anti-FLAG MAb (1/500) (M2; Sigma, Kawasaki-shi, Japan), and non-tagged human SLFN14 was detected with the antibodies anti-SLFN14 PAb (Abcam, Cambridge, UK, ab254806) (1/500) and anti-SLFN14 PAb (Invitrogen, Waltham, MA, USA, PA520868) (1/500), which recognize epitopes in the N-terminal and C-terminal regions, respectively. As a loading control, anti-α-tubulin MAb (clone B-5-1-2; Sigma) was used at a 1/4000 dilution. Membranes were incubated overnight at 4 °C with anti-FLAG and anti-SLFN14 antibodies, whereas anti-α-tubulin MAb was incubated for 30 min at 25 °C. Primary antibody-bound membranes were washed in TBS-0.1% Tween 20, and bound antibodies were detected with goat anti-mouse Ig-horseradish peroxidase (HRP) (Sigma, 1/2000) or mouse anti-rabbit IgG-HRP (Santa Cruz Biotech, Dallas, TX, USA, 1/4000) diluted in antibody dilution buffer. These antibodies were incubated for 1 h at 25 °C. Unbound secondary antibodies were washed as described above, and bound antibodies were detected by chemiluminescence.

**Analysis of SLFN14 activity.** HEK293T cells were plated at 0.45 × 10^6^ cells/well in a six-well plate, or at 10^6^ cells in a T25 flask, and transfected by calcium phosphate with the corresponding plasmids, and transfection medium was replaced with fresh culture medium 18 h later. In experiments evaluating the effect of SLFN14 on protein expression, cells were transfected in six-well plates. Each well was transfected with 1 ug of empty plasmid or 1 ug of mouse or human SLFN14 and 1 ug of the target plasmid (pNLENG1-ES-IRES, pHLuc, pCI Luc, pECFP-C1, pCMVΔR8.9, or pARP-8675), and cells were analyzed 72 h after transfection. In experiments evaluating the effect of SLFN14 on the HIV-1 infection, cells were plated in T25 flasks and transfected with 5 ug of empty plasmid or mouse or human SLFN14. Forty-eight hours after transfection, cells were detached mechanically and infected with HIV-1 (NL4-3) by spin-inoculation by resuspending cells (2 × 10^6^) in 500 μL of 37 °C-warmed culture medium in a 15 mL tube and centrifuged at 1200× *g* for 2 h at room temperature. Input HIV-1 was removed the next day by extensive washing in culture medium, and viral replication was determined by p24 ELISA at days 3 and 5 post-infection.

**Purification of primary cells.** Blood samples were obtained from two deidentified healthy individuals in concordance with approved protocol from the Institutional Review Board of the University of Texas at El Paso (ID 1741809-1, approved on 21 May 2021) and after informed consent was obtained from all subjects. All experiments were performed in accordance with relevant guidelines and regulations of our institution. Blood (60 mls) was centrifuged at 600× *g* for 10 min and plasma removed. Blood cells were mixed with 6 mls of PBS and 25 mls of this cell suspension was layered on 18 mls of Ficoll-Paque (Fisher Scientific, Hampton, NH, USA, 17144003) and spun down for 35 min at 400× *g* at room temperature. The PBMC fraction was harvested, diluted three-fold in PBS, and collected by centrifugation at 600× *g* for 10 min. Uncultured fresh PBMCs (10^6^) were lysed in 50 μL of CSKI, as described above, and analyzed by immunoblot. PBMCs (0.33 × 10^6^) were plated in round-bottom wells in 96-well plates in 100 μL of culture medium and treated with different stimuli for 72 h. In addition, PBMCs (4 × 10^6^) were subjected to isolation of naïve CD4+ T cells (MiltenyiBiotec, Bergisch Gladbach, Germany, 130-094-131) or monocytes (MiltenyiBiotec 130-096-537) following the manufacturer’s instructions, and isolated cells were plated in round-bottom wells in 96-well plates in 100 μL of culture medium and treated with different stimuli for 72 h. Treatments were culture medium (control), IL2 (30 U/mL) plus PHA (5 ug/mL), PMA (100 ng/mL), and IFN-α1 (10,000 U/mL). PBMCs and CD4+ T cells were also treated with Anti-Biotin MACSiBead (MiltenyiBiotec 130-091-441) loaded with anti-CD3 and anti-CD28 antibodies (anti-CD3/CD28 immunobeads) using one bead per two cells, and monocytes with GM-CSF (50 ng/mL) plus IL4 (50 ng/mL). Cells from one well of the 96-well tissue culture plate were lysed in 50 μL of CSKI, as described above, and 15 μL of the cell lysate was analyzed by immunoblot.

**Electroporation of immune cells.** SUP-T1 cells and primary CD4+ T lymphocytes and monocytes were electroporated using an Amaxa Cell Line Nucleofector Kit V (VCA-1003), and programs O-017 (T cells) or V-001 (monocytes) with 1 ug pNL4-3 and 1 ug empty plasmid or human SLFN14 expression plasmid. Seventy-two hours after electroporation, cell viability was determined by measuring ATP levels (Promega G9241) and 30 μL of cell supernatant was transferred to fresh SUP-T1 cells (10^5^ cells in 500 μL). HIV-1 p24 was determined by ELISA in the SUP-T1 cell culture supernatant at different times post-transfer.

**RNA polymerase III (RNA Pol III) and Janus kinase (JAK) inhibitors**. HEK293T cells were transfected as described above with empty or human SLFN14 expression plasmids and a plasmid encoding Hluc in the presence of RNA Pol III inhibitor (Sigma ML-60218, 40 and 20 uM) or tofacitinib (Sigma PZ0017, 200 and 100 nM). Transfection medium was replaced with fresh culture medium 18 h later and inhibitors were added again. Cells were analyzed 72 h after transfection.

**HIV-1 p24 ELISA.** HIV-1 p24 levels were determined by a sandwich ELISA according to the manufacturer’s instructions (ZeptoMetrix, Buffalo, NY, USA, 0801002). Briefly, cell culture supernatants were diluted appropriately and incubated on the ELISA antibody pre-coated wells overnight at 37 °C. Unbound proteins were removed by washing the wells 6 times with 200 μL of washing buffer, and bound HIV-1 p24 was detected by incubating each well with 100 μL of the anti-HIV-1 p24-HRP secondary antibody for 1 h. Unbound antibodies were removed by washing as described above, and bound antibodies were detected by incubating each well with 100 μL of substrate buffer for 30 min at room temperature until the reaction was stopped by adding 100 μL of stop solution into each well. The absorbance of each well was determined at 450 nm using a microplate reader (Versa max microplate reader; Molecular Devices).

**Luciferase assay.** Cells in suspension (100 μL, ~3 × 10^5^) were mixed with 75 μL of 0.1% Triton X-100 in PBS and 25 μL of substrate (Bright-Glow™ Luciferase Assay System, Promega), and 50 μL aliquots of the mix were distributed in triplicate wells of a 96-well white plate and analyzed in a microplate luminometer.

**Flow cytometry analysis.** CD4 and CXCR4 expression was detected in HEK293T cells (10^6^) transfected with 5 ug of empty plasmid or plasmids expressing human or mouse SLFN14 together with 5 ug of plasmids expressing human CD4 and CXCR4. Transfected cells were harvested by mechanical dissociation and 10^5^ cells re-suspended in 100 μL of 1x PBS containing 1 μL of Alexa 488-labeled anti-human CD4 (eBioscience™, 53-0048-42) and incubated on ice for 5 min. CXCR4 and mCherry are expressed from the same bicistronic plasmid, and therefore mCherry was used as a proxy of CXCR4 transfection efficiency. Furthermore, to evaluate the effect of SLFN14 on the expression of LTR-driven eGFP, HEK293T cells were co-transfected with plasmids expressing HIVeGFP [56] and cyan fluorescence protein (transfection control), together with either control (empty) or SLFN14-expression plasmids, and analyzed 72 h later by flow cytometry. In all these experiments, cells were analyzed with a Gallios flow cytometer (Beckman Coulter), and fluorescence minus one controls were used to set up the flow cytometer. Data were analyzed with Kaluza Analysis software version 1.3.

**Quantitative RT-PCR and PCR, and rRNA integrity analyses.** Total RNA was isolated from cells or sucrose density gradient fractions using TRIzol LS reagent (Invitrogen 10296010) according to the manufacturer’s instructions. All RNA samples had ratios of absorbance at 260/280 nm of 1.8 to 2.0, indicating that samples were contaminant-free and that the RNA integrity number equivalent (RIN^e^) > 9.0. Purified RNA samples were stored at −80 °C until use. Different mRNAs were detected by RT-PCR (BioRad, Hercules, CA, USA, 1725151) using 1 ng of total RNA. HIV-1 Gag wild-type mRNA was detected with primers DR22 (5′-agcaggaactactagtaccc-3′) and DR23 (5′-ttgtcttatgtccagaatgc-3′), while primers CV35 (5′-cgccggcaccacaagcaccc-3′) and CV36 (5′-ctgcttgatgtccaggatgc-3′) were used to detect codon-optimized Gag, and primers EL9 (5′-acccctggccaaggtcatcc-3′) and EL10 (gacggcaggtcaggtccacc) were used for GAPDH. Human SLFN14 was detected by RT-PCR using primers CV43 (5′-atggatgttttcagccttccactaaggatttgc-3′) or SS3 (5′-GCTCCTTCCTTCAGGTTCACAG-3′) and SS1 (5′-atccaccccaatgaggacatatcc-3′) that bind to exon 3, which encodes the N-terminal region of the protein. DNA was extracted from cells transfected with pCMVΔR8.91 using TRIzol LS reagent and PCR amplified with primers DR22 and DR23 using IQ SYBR Green Supermix (BioRad, 1708882). Total RNA from 0.33 × 10^6^ cells was used for electrophoretic analysis of rRNA integrity with an Agilent Technologies 4200 TapeStation. As previously reported [57], RNA degradation bands were considered to be those migrating between the 18S rRNA and small RNA bands in the region of 1500 base pairs (bp) to 200 bp. RNA degradation bands were quantified with the Agilent TapeStation Controller Software 4.1.

**Sucrose Density Gradient.** HEK293T cells (3 × 10^6^) were plated in a T25 and calcium phosphate transfected with 4 ug of empty plasmid or human SLFN14 expression plasmid and 4 ug of pCMVΔR8.91 or pARP-8675. The next day, the transfection medium was replaced with fresh culture medium and, 48 h later, cells were mechanically harvested and used for the sucrose density gradient (SDG), as previously described [41,42]. Briefly, cells were lysed by incubation in ice in Buffer A (20 mM Tris-HCl, pH 7.5, 100 mM KCl, 2.5 mM MgCl_2_, 1 mM DTT, 0.25 mM spermidine) supplemented with 0.5% Triton X-100 and protease inhibitors (final concentrations of 2 μg/mL leupeptin, 5 μg/mL aprotinin, 1 mM phenylmethylsulfonyl fluoride, and 1 μg/mL pepstatin A). Cell lysates were centrifuged at 2000× *g* for 10 min at 4 °C and the supernatant loaded on top of a 15 mL 10–30% SDG prepared in buffer A supplemented with 0.1 mg/mL cycloheximide and protease inhibitors. Cycloheximide was used to prevent polysome runoff [42], SDG was centrifuged at 117,100× *g* for 3.5 h at 4 °C in a Sorvall WX 80+ Ultracentrifuge in a Surespin 630 rotor in 17 mL tubes (Thermo, 79386). Fractions (500 μL) were collected from top to bottom of the gradient (30 fractions). SLFN14 was detected by immunoblotting with an anti-FLAG antibody in each fraction, loading per lane 12 μL, whereas RNA was extracted from 400 μL of the fraction with TRIzol LS reagent, as described above.

**In silico analysis.** The codon adaptation index was determined with the CAIcal program [http://genomes.urv.cat/CAIcal, accessed on 11 December 2022 [58]], as described in [59], using as reference the human codon usage table (http://genomes.urv.cat/CAIcal/CU_human_nature, accessed on 11 December 2022). SLFN14 molecular weight was predicted using Expasy. Biorender software was used to generate the graphical abstract.

**Statistical Analysis.** GraphPad Prism version 9.4.1 was used for statistical analysis. One-way ANOVA was used to test the impact of human and mouse SLFN14 on the expression of the proteins of interest, and the Dunnett’s post hoc test was used to identify significant differences between cells expressing empty plasmid (control group) and cells expressing SLFN14 proteins (experimental groups). A two-tailed, two-sample *t* test was used to evaluate the statistically significant of experiments with only two groups (control and experimental). In experiments where the comparison was between a specific control and a specific experimental group, one-way ANOVA and the Bonferroni post hoc test was utilized. *p*-values were indicated as follows: not significant (ns) > 0.05, * ≤ 0.05, ** ≤ 0.01, *** ≤ 0.001, **** ≤ 0.0001.

**Accession numbers:** The sequences of the 5′end of the SLFN14 exon 3 that we detected in MOLT-3 cells is deposited under OP548624 and OP548623.

## 3. Results

### 3.1. Human and Mouse SLFN14 Preferentially Impairs the Expression of Proteins Enriched in Rare Codons

To evaluate the effect of SLFN14 on the expression of genes enriched in rare codons, we used HIV-1 as a model. Advantageously, HIV-1 gene expression can be studied with plasmids encoding this virus or its individual proteins, or using the copy of the viral genome integrated into the host chromosome, whose expression is regulated as any cellular gene is. Furthermore, HIV-1 has open reading frames with different codon usage that are included in a common transcript produced from the viral promoter. Moreover, different reporter genes can be efficiently inserted into the HIV-1 genome. To study HIV-1 protein expression, we used the methodology described before to determine the anti-HIV-1 activity of SLFN11 [31,32], SLFN13 [38], and N4BP1 [60]. This method has been also extensively used to evaluate the production phase of the HIV-1 life cycle. In this experimental strategy, HEK293T cells are co-transfected with plasmids expressing the proteins being evaluated and a plasmid encoding HIV-1. Because HEK293T cells lack detectable expression of SLFN14 mRNA [41] these cells are suitable for studying SLFN14.

To analyze the activity of SLFN14, we used an HIV-1 reporter (HIVeGFP, [56]) that expresses from the viral promoter, in a Tat-dependent manner, a transcript that contains open reading frames with synonymous codon usage that greatly differ from the host, such as Gag (CAI of 0.56). A fraction of this transcript experiences splicing, generating other transcripts containing open reading frames whose synonymous codon usage resemble the host preferences, for example, Tat (CAI of 0.761), and a reporter gene (eGFP) that is codon-optimized for human cells (CAI of 0.962). Therefore, this reporter offers the possibility to evaluate the effect of SLFN14 on the expression of transcripts with three different CAIs.

**Figure 1:**
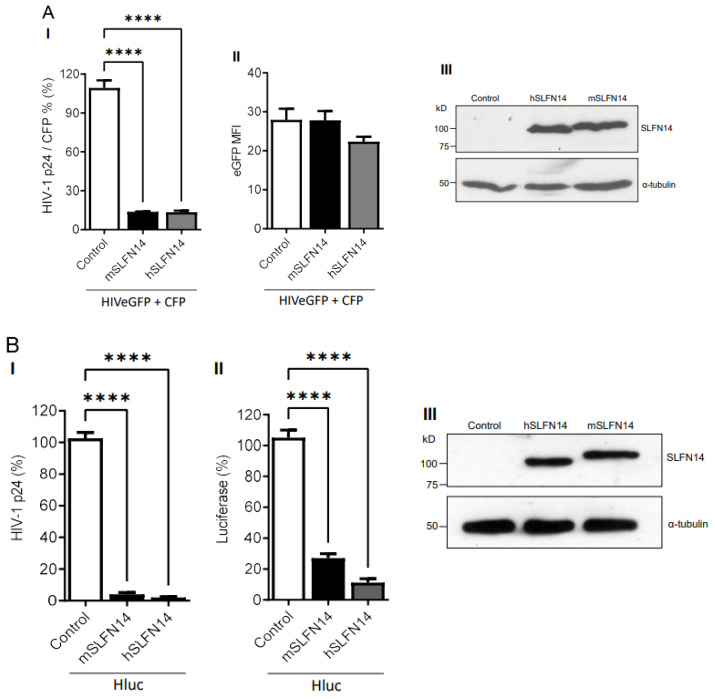
**Effect of SLFN14 on HIV-1-driven protein expression.** (**A**). HEK293T cells that were co-transfected with a plasmid encoding an HIV-1 expressing eGFP and a plasmid expressing CFP, and either an empty plasmid (control cells) or a plasmid expressing human (h) or mouse (m) SLFN14. (**I**) HIV-1 p24 levels were normalized for transfection efficiency (% of CFP+ cells) and expressed as % of control cells. (**II**) eGFP MFI levels and (**III**) SLFN14 protein expression in cells represented in panel (**I**). In the immunoblot (**III**), an anti-FLAG antibody was used to detect SLFN14, and α-tubulin levels were determined as a loading control. Data correspond to a triplicate experiment and are representative of five independent experiments. (**B**) HIV-1 p24 (**I**) and luciferase (**II**) levels expressed as % of control cells in HEK293T cells that were co-transfected with a plasmid encoding an HIV-1 expressing firefly luciferase (Hluc), and either an empty plasmid (control cells) or a plasmid expressing human (h) or mouse (m) SLFN14. (**III**) Expression of SLFN14 in these cells was detected by immunoblotting as described in (**A-III**). Data correspond to a triplicate experiment and are representative of more than twenty independent experiments performed along several months. Statistically significance in (**A**,**B**) was calculated with one-way ANOVA and Dunnett post hoc tests. **** *p* ≤ 0.0001. Data showing not statistically significant differences (NS *p* > 0.05) are not indicated in any of the figures of this work. Figure is created by Valenzuela et al.

HEK293T cells co-transfected with plasmids expressing HIVeGFP [56] and cyan fluorescence protein (CFP, transfection control), together with either an empty plasmid (control) or plasmids expressing mouse or human SLFN14 were analyzed. Data in Figure 1A(I) show that cells expressing either mouse or human SLFN14 produced ~86% less HIV-1 p24 (a limited proteolysis product of Gag) than control cells. HIV-1 p24 values shown were normalized to the % of CFP+ cells to account for transfection efficiency. Despite this, severe reduction in HIV-1 p24, the HIV-1-driven eGFP expression, as determined by the eGFP Mean Fluorescence Intensity (MFI), an estimate of the number of eGFP molecules per cell, was similar in cells expressing or no SLFN14 proteins (Figure 1A(II)). SLFN14 levels were verified in these cells by immunoblot (Figure 1A(III)). 

Because the HIV-1-driven transcript encoding eGFP derives from the messenger encoding Gag, preservation of eGFP expression indicated that SLFN14 impaired Gag production at a post-transcriptional step. Furthermore, since, in this model, HIV-1 transcription depends on Tat, the levels of this protein were not significantly diminished by SLFN14 proteins. Therefore, SLFN14 proteins selectively impaired the expression of Gag, the lower CAI protein in the group analyzed. The similar activity of the mouse and human proteins was expected since they are 70% identical, representing the highest evolutionary conservation between human SLFNs and their mouse orthologs in the SLFN group III [61].

Next, we evaluated the effect of SLFN14 on the expression of a different HIV-1 reporter (Hluc, [50]) that produces firefly luciferase (CAI of 0.71) instead of eGFP. As before, HEK293T expressing mouse and human SLFN14 produced ~97% less HIV-1 p24 than the control cells (Figure 1B(I)), whereas luciferase expression was reduced by ~73% and ~89% by mouse and human SLFN14, respectively (Figure 1B(II)). SLFN14 levels were verified in these cells by immunoblot (Figure 1A(III)). These findings further demonstrated that SLFN14 impairs the expression of viral and non-viral proteins with low CAIs.

**Figure 2:**
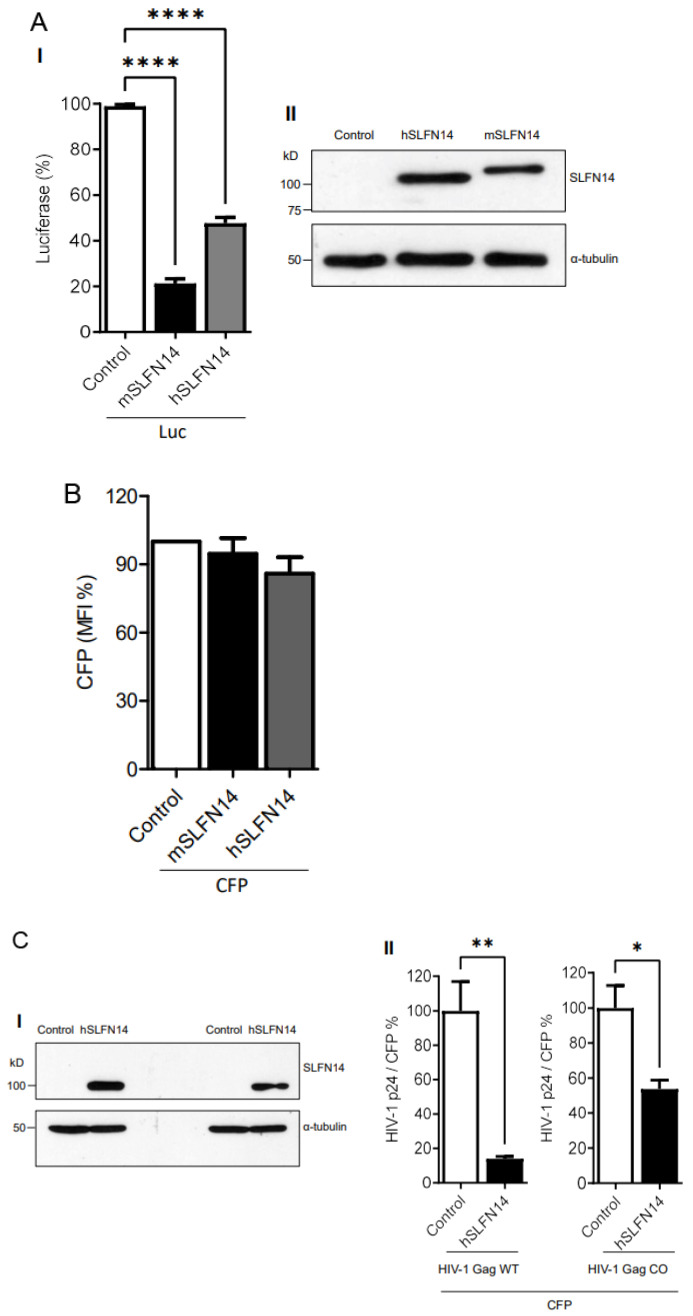
**Effect of SLFN14 on the expression of transcripts with different codon usage**. HEK293T cells were co-transfected with a plasmid expressing firefly luciferase or CFP and either an empty plasmid (control cells) or a plasmid expressing human (h) or mouse (m) SLFN14. Luciferase activity (**A-I**) and CFP MIF (**B**) were expressed as % of control cells. (**A-II**) Expression of SLFN14 in these cells was detected as described in Figure 1A(III). Statistical significance was determined by one-way ANOVA and Dunnett post hoc tests. **** *p* ≤ 0.0001. Data correspond to a triplicate experiment and are representative of five (**A-I**) or six (**B**) independent experiments. (**C**) Effect of SLFN14 on HIV-1 Gag wild-type (WT) and codon-optimized (CO) expression. HEK293T cells were co-transfected with a plasmid expressing CFP and plasmids expressing either HIV-1 Gag WT or COtogether with either an empty plasmid or an SLFN14 expression plasmid. (**I**) Expression of SLFN14 in these cells was detected as described in Figure 1A(III). (**II**) HIV-1 p24 was normalized for transfection efficiency (% of CFP+ cells) and expressed as % of control cells. Statistically significant differences were calculated with repeated measures using a two-tailed, two sample *t* test. ** *p* ≤ 0.01 and * *p* ≤ 0.05. Data correspond to a triplicate experiment and are representative of eight independent experiments. Figure is created by Valenzuela et al.

Using the cellular model described above, we also determined the effect of SLFN14 proteins on the expression of non-viral proteins with different CAIs using a non-HIV-1 and simpler gene expression system based in the CMV enhancer/promoter. SLFN14-transfected cells produced ~79% (mouse) and ~53% (human) less firefly luciferase than the control cells (Figure 2A(I)); however, CFP (CAI of 0.96) MFI was not reduced (Figure 2B). These findings further supported that SLFN14 selectively impairs expression of transcripts enriched in rare codons. 

To further evaluate a potential function of SLFN14 in codon usage-based control of gene expression, we determined the effect of SLFN14 on the expression of HIV-1 Gag, wild-type and codon-optimized (CAI of 0.99). These Gag open reading frames were under the transcriptional control of the CMV enhancer/promoter. In these experiments, HEK293T cells were co-transfected with plasmids expressing CFP and wild-type or codon-optimized Gag and SLFN14 proteins, and HIV-1 p24 levels were normalized to the percentage of CFP positive cells to account for transfection efficiency. Although similar levels of SLFN14 were achieved in these cells (Figure 2C(I)), SLFN14 impaired by ~10 folds the expression of wild-type Gag and by only ~1.8 folds the expression of codon-optimized Gag (Figure 2C(II)), indicating that SLFN14 preferentially impairs expression of transcripts enriched in rare codons.

### 3.2. Expression of SLFN14 in Cells of the Immune System

**Figure 3:**
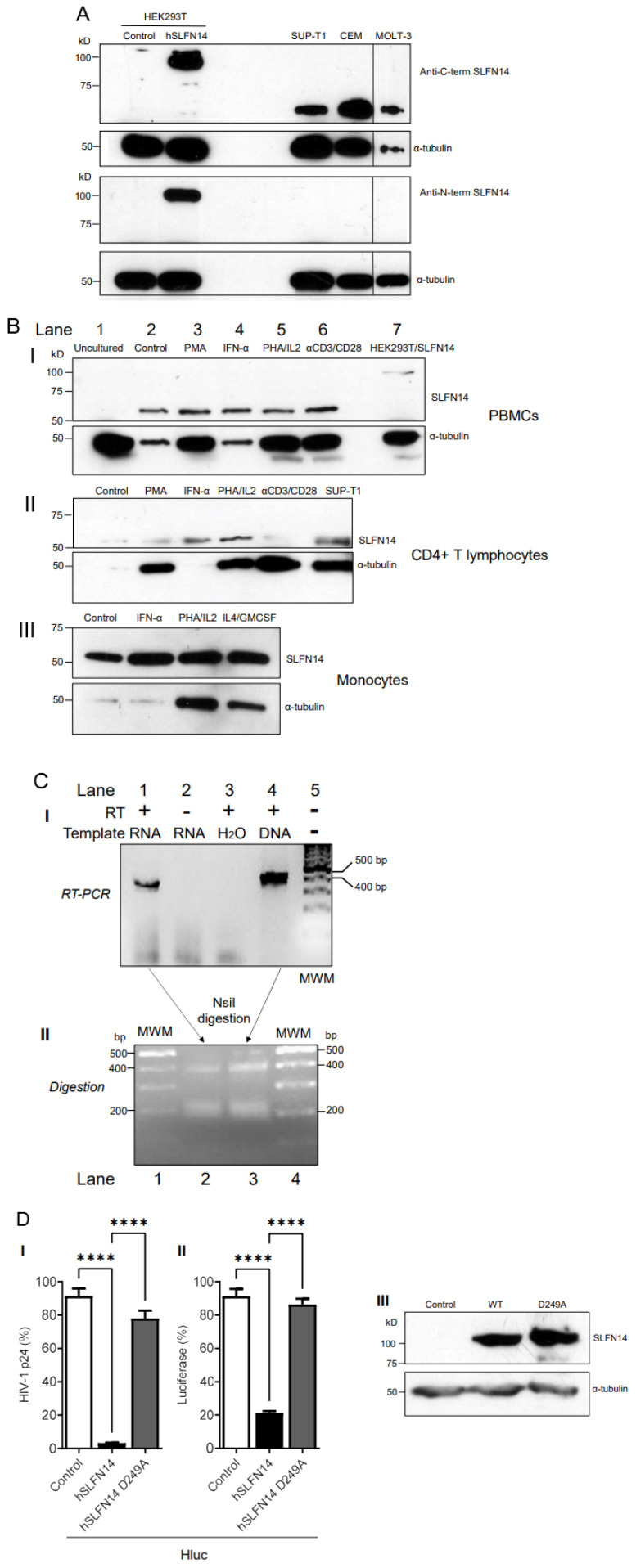
**Expression of SLFN14 in immune cells.** (**A**). Immunoblot analysis of SLFN14 expression in HEK293T co-transfected with an empty plasmid (Control) or a plasmid expressing human SLFN14, and in non-transfected SUP-T1, CEM, and MOLT-3 cells. The MOLT-3 cell lysate was in the same immunoblot membrane than the other samples but separated by irrelevant samples that were removed from the image presented. Anti-SLFN14 antibodies recognizing the N- or the C-terminus of the protein were used as indicated, and α-tubulin levels were determined as a loading control with a specific antibody. (**B**) Immunoblot analysis of SLFN14 expression in PBMCs (**I**), and PBMC-derived CD4+ T lymphocytes (**II**), and monocytes (**III**) with an anti-C-terminal SLFN14 antibody. α-tubulin was determined as a loading control. Cells were subjected to the stimuli indicated. Positive controls were HEK293T cells co-transfected with human SLFN14 (HEK393T/SLFN14) and SUP-T1 cells. (**C**) Analysis of SLFN14 mRNA in MOLT-3 cells. (**I**). Agarose gel electrophoresis analysis of the reverse transcription (RT)-PCR evaluating SLFN14 expression in MOLT-3 cells with primers SS1 and CV43. Samples were loaded in the gel in the following order: Lane 1 RT-PCR with RNA, Lane 2 No RT control (PCR with RNA), Lane 3 No template control (RT-PCR with no RNA), Lane 4 Positive control (RT-PCR with SLFN14 expression plasmid), and Lane 5 DNA Molecular Weight Marker (MWM). (**II**). Agarose gel electrophoresis analysis of the digestion with NsiI of the RT-PCR products obtained with primers SS1 and CV43 using as template RNA extracted from MOLT-3 cells (lane 2) or the SLFN14 expression plasmid (lane 3). DNA molecular weight markers (MWM) are in lanes 1 and 4. (**D**) Effect of D249A mutation on the activity of human (h) SLFN14 on transgene expression. HEK293T cells were co-transfected with a plasmid encoding an HIV-1 expressing firefly luciferase and either, an empty plasmid (control cells) or a plasmid expressing human SLFN14 WT or D249A mutant. HIV-1 p24 levels (**I**) and luciferase activity (**II**) were expressed as % of control cells. (**III**) SLFN14 expression was validated by immunoblotting as described in Figure 1A(III). Statistically significant differences were calculated with two-way ANOVA and Dunnett post hoc tests. **** *p* ≤ 0.0001. Data correspond to a triplicate experiment and are representative of three independent experiments. Figure is created by Valenzuela et al.

Since SLFN14 potently impairs HIV-1 Gag expression, we predicted that this protein should not be very abundant in cells naturally permissive to HIV-1. Therefore, we characterized the expression of endogenous SLFN14 in cells of the immune system by immunoblot. In this analysis, we used the only two anti-SLFN14 antibodies commercially available. These antibodies recognize the N-terminal (residues 100–250) and the C-terminal (amino acids 730–780) regions of SLFN14. As control, we analyzed HEK293T, since SLFN14 mRNA was reported undetectable in these cells [41]. HEK293T cells were transfected with an empty plasmid or a plasmid expressing human SLFN14, and seventy-two hours later analyzed by immunoblot. As expected, no endogenous SLFN14 was detected in these cells with any of the anti-SLFN14 antibodies evaluated, although both recognized the exogenous SLFN14 (Figure 3A).

In addition, SUP-T1 cells (lymphoblastic lymphoma-derived CD4+ T cell line), and the acute lymphoblastic leukemia-derived CD4+ T cell lines CEM and MOLT-3, were evaluated by immunoblot with these antibodies. Data in Figure 3A indicate that the anti-C-terminal SLFN14 antibody (I), but not the antibody directed against the N-terminus (II), reacted with a band that migrated slightly above 50 kD in the three cell lines, suggesting the expression of an N-terminal truncated form of the protein.

We also analyzed the expression of SLFN14 in primary immune cells. PBMCs were isolated by sedimentation on a Ficoll-Paque and lysed, and proteins were analyzed by immunoblot with anti-SLFN14 antibodies recognizing the C- and the N-terminus. No SLFN14 protein was detected in freshly isolated, uncultured PBMCs (Figure 3B(I) lane 1).

Furthermore, we explored the effect of different stimuli on the expression of SLFN14 in primary cells. Equal numbers of PBMCs (Figure 3B(I)), CD4+ T cells (Figure 3B(II)), or monocytes (Figure 3B(III)) were treated with culture medium alone (Control, Figure 3B(I) lane 2, B(II), B(III) lane 1) or supplemented with phorbol myristate acetate (PMA, Figure 3B(I) lane 4, B(II) lane 2), interferon α1 (IFN-α1, Figure B(I) lane 4, B(II) lane 3, B(III) lane 2), phytohemagglutinin and interleukin-2 (PHA/IL2 Figure 3B(I) lane 5, B(II) lane 4, B(III) lane 3), anti-CD3/-CD28 antibody-coated beads (αCD3/CD28, Figure 3B(I) lane 6, B(II) lane 5), or interleukin-4 and granulocyte-macrophage colony-stimulating factor (IL4/GMCSF, Figure 3B(III) lane 4).

After three days of treatment, all the cells in the culture were lysed in the same volume, and equal volumes of the cell lysates were evaluated by immunoblot. Notice that rather than equal protein amounts, the same number of initial cells in the culture were loaded per electrophoresis lane in these immunoblots. This strategy, combined with the detection of α-tubulin by immunoblot, additionally allowed verifying the effect of the treatments on cellular proliferation. As positive controls in these immunoblots, HEK293T cells transfected with SLFN14 (Figure 3B(I) lane 7) and SUP-T1 cells (Figure 3B(II) lane 6) were analyzed.

In contrast to fresh PBMCs that did not express any form of SLFN14 (Figure 3B(I) lane 1), after three days of in vitro culture, PBMCs expressed a 50 kD protein reactive only with the anti-C terminal SLFN14 antibody (Figure 3B(I) lane 2). The size of this band was similar to the one detected in CD4+ T cell lines (Figure 3A,B(II) lane 6) and in CD4+ lymphocytes (Figure 3B(II) lane 1) and monocytes (Figure 3B(III) lane 1). The anti-N-terminal SLFN14 failed to recognize any protein in any of the primary cell lysates.

PMA, PHA/IL-2, and anti-CD3/-CD28 immunobeads induced robust cellular proliferation in PBMCs (Figure 3B(I)) and CD4+ T cells (Figure 3B(II)), as indicated by the α-tubulin levels detected in these cultures, compared to the untreated cultures (control). Similarly, PHA/IL-2, and IL-4/GMCSF induced cellular proliferation in monocytes (Figure 3B(III)). Considering α-tubulin levels as a proxy of the number of cells analyzed, stimuli that induced cell proliferation also decreased the expression of SLFN14 per cell (Figure 3B). In contrast, IFN-α1 increased the levels of the 50 kD anti-C-terminal SLFN14 antibody-reactive protein in CD4+ T cells (Figure 3B(II)) but not in PBMCs or monocytes (Figure 3B(I),B(III)).

To analyze a potential mechanism implicated in the lack of expression of full-length SLFN14 in MOLT-3 cells, we determined whether these cells express an SLFN14 mRNAs carrying nucleotides 386–788 of exon 3 that encode amino acids 115–248. This protein region contains the epitope recognized by the anti-N-terminal SLFN14 antibody and that seems to be missing in the 50 kD anti-C-terminal SLFN14 antibody-reactive protein. RT-PCR analysis indicated robust expression of mRNAs harboring this region of exon 3 (Figure 3C(I)). The identity of the RT-PCR product was determined by overlapping DNA sequencing (GenBank accession numbers OP548624 and OP548623), and by restriction digestion with NsiI (Figure 3C(II)). This enzyme is predicted to split the 402 bp RT-PCR product in 215 and 187 bp bands. In this experiment, partial digestion was obtained as indicated by the presence of the 402 bp RT-PCR product and a thick ~200 bp band that we interpreted as the 215 and 187 bp bands closely migrating. Similar results were obtained with another set of primers targeting nucleotides 302–788 that encodes amino acids 86–248 in SLFN14. These findings are in agreement with the reported exon/intron organization of SLFN14 (NM_001129820.2), excluding alternative splicing as a mechanism in the generation of the proposed N-terminal deleted form of SLFN14 detected in immune cells. Therefore, a post-splicing event seems to determine the absence of full-length SLFN14 in these cells.

### 3.3. SLFN14 Requires the Endoribonuclease Activity to Impair HIV-1 Expression

Protein molecular weight prediction (Expasy) indicated that the SLFN14 full-length protein would be 104 kD, as evidenced by our immunoblots (for example Figure 3A). A SLFN14 protein lacking the first 330 or 420 amino acids is estimated to be 69 or 56 kD, respectively, being in the molecular weight range of the N-terminal-truncated form of SLFN14 detected in immune cells (Figure 3A,B). These predicted that N-terminal-truncated SLFN14 proteins would lack the epitope recognized by the anti-N-terminal antibody (residues 100–250), including the residue 249, which is essential for the endoribonuclease activity [42].

Since cells expressing the N-terminal truncated form of SLFN14 are susceptible to HIV-1, we predicted that SLFN14 requires the endoribonuclease activity to repress expression of HIV-1 Gag. Therefore, we determined the anti-HIV-1 activity of an SLFN14 endoribonuclease-dead mutant (D249A) [42] in HEK293T cells co-transfected with plasmids expressing SLFN14 wild type or the D249A mutant and an HIV-1 reporter that expresses LTR-driven luciferase [50]. In concordance with our previous observations, SLFN14 wild type decreases HIV-1 p24 (Figure 3D(I)) and luciferase (Figure 3D(II)) levels by approximately 98% and 80%, respectively. However, this inhibitory activity was drastically reduced by the D249A mutation, despite the wild-type and mutant proteins being expressed at similar levels (Figure 3D(III)). Therefore, the endoribonuclease activity of SLFN14 is required for the role of this protein in gene expression regulation, and, likely, the N-truncated form of the protein expressed in HIV-1-permissive immune cells is inactive.

### 3.4. SLFN14 Impairs HIV-1 Protein Expression in CD4+ T Cells and Monocytes

To evaluate the effect of SLFN14 on HIV-1 protein expression in HIV-1-permissive immune cells, SUP-T1 cells and primary CD4+ lymphocytes and monocytes were electroporated with wild-type HIV-1 (pNL4-3) and human SLFN14 or empty plasmids, and three days later, the cell supernatant was used to infect fresh SUP-T1 cells.

**Figure 4:**
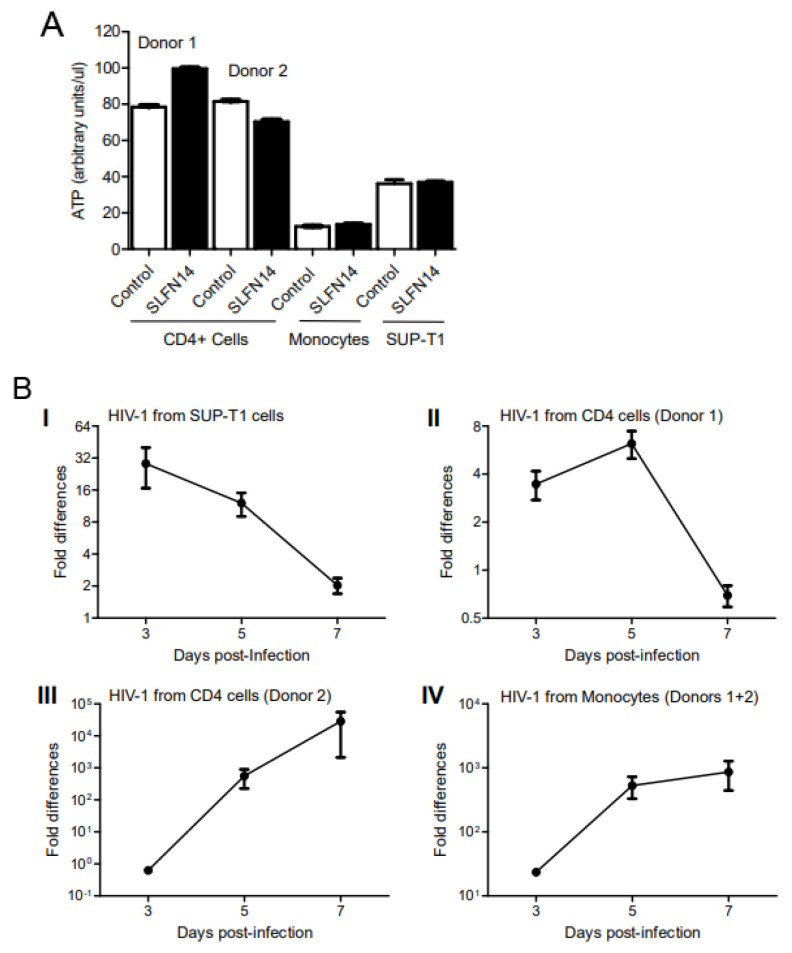
**Effect of SLFN14 on HIV-1 production in immune cells**. CD4+ T lymphocytes from two donors, pooled monocytes from these donors, and three independent SUP-T1 cell cultures were electroporated with a plasmid expressing HIV-1 wild type and either an empty plasmid (control) or a plasmid expressing human SLFN14. Seventy-two hours later, ATP levels were quantified in triplicate measurements of the electroporated cells (**A**), and cell supernatant from the electroporated cells was transferred in triplicate to fresh SUP-T1 cell cultures. HIV-1 replication in these SUP-T1 cells was followed by measuring HIV-1 p24 levels in the cell supernatant. (**B**) Fold differences in HIV-1 p24 levels in SUP-T1 cells infected with the virus produced in electroporated SUP-T1 cells (**I**), CD4+ lymphocytes from donor 1 (**II**) or donor 2 (**III**), and pooled monocytes from donors 1 and 2 (**IV**). Statistical analysis was conducted in by two-tailed, two-sample *t* test. Figure is created by Valenzuela et al.

Although similar cell viability was observed in the electroporated HIV-1 producer cells, as indicated by their ATP content (Figure 4A), HIV-1 production was severely impaired by SLFN14 (Figure 4B). Three days after infection, HIV-1 p24 levels were 28- and 3-fold higher in cells infected with control than SLFN14 viruses produced in SUP-T1 and CD4+ T lymphocytes (donor 1), respectively (Figure 4B(I),B(II)). Levels of HIV-1 p24 similar to those detected in cells infected with viruses from SUP-T1 and CD4+ T lymphocytes (donor 1) at day 3 were observed in cells infected with viruses from CD4+ T lymphocytes (donor 2) cells and pooled monocytes only at day 5 post-infection. At this time point, 561- and 23-fold more HIV-1 p24 was produced in cells infected with control than SLFN14 viruses produced in CD4+ T lymphocytes (donor 2) and pooled monocytes, respectively (Figure 4B(III),B(IV)). Control HIV-1-infected SUP-T1 cultures that showed earlier HIV-1 p24 production (Figure 4B(I),B(II)) succumbed earlier to the infection, reducing the differences observed at day 3 post-infection. In these cultures, levels of HIV-1 p24 were between 500–1000 ng/mL at day 7 post-infection. In contrast, SUP-T1 cultures where infection was manifested at a later time point (Figure 4B(III),B(IV)) did not exhibit significant cytopathic effects at day 7 post-infection, producing between 1 and 80 ng/mL HIV-1 p24. 

The stronger anti-HIV-1 activity of SLFN14 observed in CD4+ T lymphocytes from donor 2 as compared to cells from donor 1 could have been due to a higher electroporation efficiency of the cells from donor 1. These differences would explain the more robust HIV-1 replication observed at early (day 3) and late (day 7) stages of infection in the reporter SUP-T1 cells infected with virus produced in CD4+ T lymphocytes from donor 1. The postulated lower electroporation efficiency of CD4+ T lymphocytes from donor 2 could have affected more HIV-1 production than the anti-viral effect of SLFN14, due to the high potency of SLFN14. Then, the proposed combination of low levels of HIV-1 mRNA and sufficient, although low, levels of SLFN14 protein could have determined the stronger anti-HIV-1 activity of SLFN14 observed in CD4+ T lymphocytes from donor 2.

In sum, findings in Figure 4 demonstrated that SLFN14 can also inhibit HIV-1 expression in cell types that are relevant in vivo to this virus.

### 3.5. Effect of SLFN14 on Viral Infection

Experiments reported above showed that SLFN14 impairs expression of rare codon-enriched transcripts expressed from transfected plasmids. Since SLFN14 affects preferentially the expression of wild-type (AT-rich) rather than codon-optimized (AT-poor) Gag, the RNA Pol-III-RIG-I pathway could be implicated. RNA Pol III transcribes AT-rich DNA templates producing short AU-rich RNA fragments that induce type I IFN via RIG-I [62]. This innate immune pathway is active in HEK293T cells [63]. Furthermore, transfection of HEK293T cells with in vitro transcribed HIV-1 wild-type-encoded RNAs has been reported to trigger type I IFN, and this effect was inhibited by codon optimization of the viral sequences to resemble the human codon usage [64].

**Figure 5:**
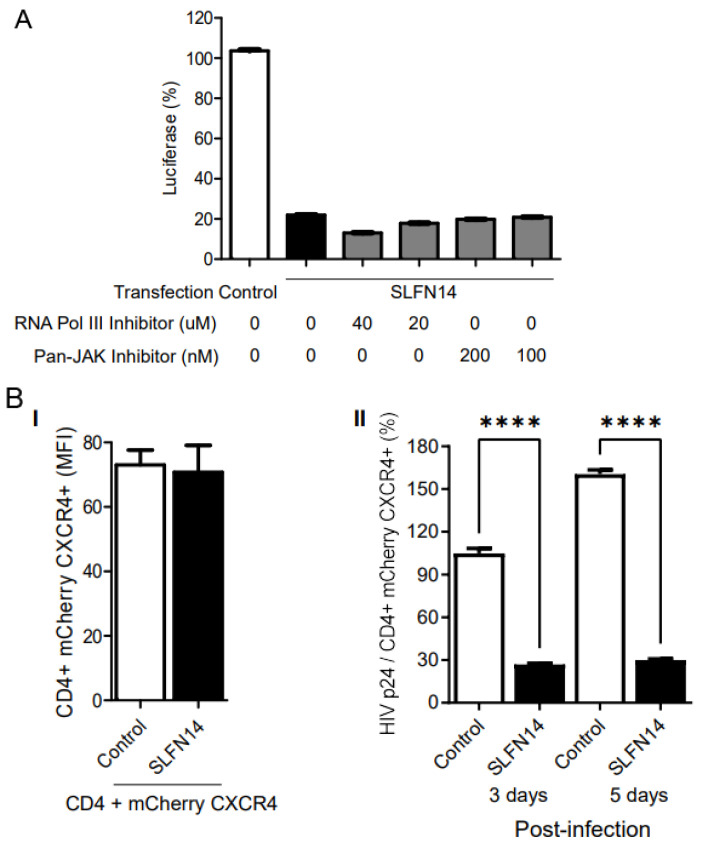
**Effect of SLFN14 on viral replication.** (**A**) Role of the RNA Polymerase III-RIG-I-IFN signaling pathway in the SLFN14 activity. HEK293T cells co-transfected with HIVluc expression plasmid and an empty plasmid (control cells) or a plasmid expressing human SLFN14 were treated or not with inhibitors indicated. Luciferase activity measured in these cells was expressed as % of control cells. Data correspond to a triplicate experiment and are representative of three independent experiments. Although not indicated, statistically significant differences (*p* ≤ 0.0001) were found between the control and each of the other groups, as calculated with two-way ANOVA and Dunnett post-hoc tests. (**B**) Effect of SLFN14 on HIV-1 replication. (**I**) CD4 and CXCR4 expression. HEK293T cells were co-transfected with plasmids expressing CD4 and a bicistronic plasmid encoding CXCR4 and mCherry, and either, an empty plasmid (control cells) or a human SLFN14 expression plasmid. CD4 was detected by immunostaining and MFI values of CD4 and mCherry (CXCR4) were quantified by flow cytometry. (**II**) These cells were infected with HIV-1 wild-type and viral replication was followed by measuring HIV-1 p24 in the cell supernatant. Data pertain to a triplicate experiment and they are representative of two independent experiments. Statistically significant differences were calculated with one-way ANOVA and Bonferroni post-hoc tests **** *p* ≤ 0.0001. Figure is created by Valenzuela et al.

To investigate the implication of type I IFN signaling in the inhibitory effect of SLFN14 on gene expression, we transfected HEK293T cells with plasmids expressing an LTR-driven luciferase HIV-1 reporter [50] and either empty or SLFN14-encoding plasmid. Transfection was performed in the presence of RNA polymerase III or pan-Janus kinase inhibitors, and these inhibitors were kept during the entire duration of the experiment. Under these conditions, SLFN14 reduced by 80% the expression of luciferase found in control cells, and this effect was not modified by RNA polymerase III or pan-Janus kinase inhibitors (Figure 5A). These findings excluded a role of the RNA Pol-III-RIG-I-IFN pathway in the activity of SLFN14.

Next, we evaluated the effect of SLFN14 on the expression of rare codon-enriched transcripts expressed by viral infection. HEK293T cells co-transfected with plasmids expressing the HIV-1 receptor (CD4) and co-receptor (CXCR4), with plasmids either empty or encoding SLFN14, were infected with HIV-1 NL4-3. Ectopic expression of the HIV-1 receptor has been successfully used previously to study different aspects of HIV-1 biology [for example, [65,66,67]]. In these experiments CXCR4 was expressed from a bicistronic plasmid together with mCherry; therefore, levels of the fluorescence protein served as a surrogated of CXCR4 expression and transfection efficiency.

Surface CD4 and CXCR4 expression was demonstrated to be equivalent at the time of infection in control or SLFN14 cells by flow cytometry, and cell supernatant HIV-1 p24 levels were normalized to the % of CD4/mCherry CXCR4 double positive cells. The MFI of CD4 and mCherry was similar in cells transfected with control and SLFN14 expression plasmids (Figure 5B(I)), indicating that SLFN14 did not affect the expression levels of CD4 or mCherry that exhibit CAIs of 0.82 and 0.976, respectively. However, cells expressing SLFN14 produced approximately ~73% and ~83% less HIV-1 p24 than control cells at days 3 and 5 post-infection, respectively (Figure 5B(II)). HIV-1 p24 at day zero was undetectable. These findings demonstrated that SLFN14 can impair expression of Gag from an integrated provirus.

### 3.6. SLFN14 Caused Ribosomal RNA Degradation in Cells Co-Expressing Gag Wild Type

SLFN14 is a ribosome-associated endoribonuclease [41,42]. Purified SLFN14 was reported to degrade purified ribosomal, tRNA, and mRNA in vitro [42]. In addition, our results here indicate that SLFN14 restricts the expression of transcripts rich in rare codons at a post-transcriptional step and requiring the endoribonuclease activity. Therefore, we sought to evaluate the effect of SLFN14 on rRNA and mRNA levels. In these studies, we used as a model wild-type and codon-optimized Gag, expecting to identify a SLFN14-dependent mechanism that operates preferentially in the cells expressing wild-type Gag.

HEK293T cells were co-transfected with plasmids expressing HIV-1 Gag, wild-type or codon-optimized, together with a plasmid expressing firefly luciferase (pCI Luc), and either empty plasmid or plasmids expressing human or mouse SLFN14.

**Figure 6:**
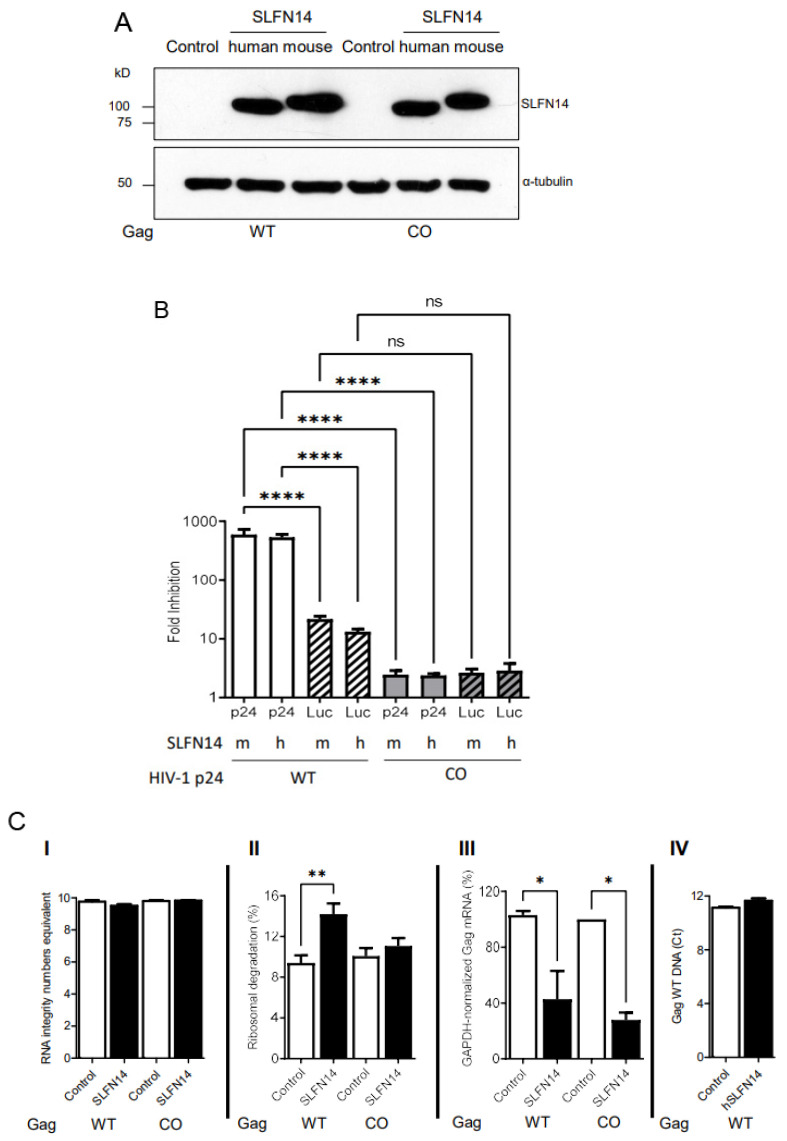
**Effect of SLFN14 on nucleic acid integrity.** HEK293T were co-transfected with a plasmid expressing luciferase (Luc), a plasmid encoding Gag, wild-type (WT) or codon-optimized (CO), and either an empty plasmid (control cells) or a plasmid encoding SLFN14, human or mouse. (**A**) SLFN14 expression was verified by immunoblot as described in Figure 1A(III). (**B**) HIV-1 p24 levels and luciferase activity determined in these cells were expressed as fold inhibition relative to control cells. Note that the Y axes of the graphic is in log10 scale. Data correspond to a triplicate experiment that is representative of five independent experiments. Statistically significant differences were calculated using one-way ANOVA and a Tukey pos hoc test. (**C**) Effect of SLFN14 on nucleic acids. RNA quality (**I**), ribosomal RNA degradation (**II**), GAPDH mRNA-normalized Gag mRNA levels (**III**), and Gag WT cDNA levels (**IV**) were expressed relative to values found in control cells. Data correspond to a triplicate experiment and are representative of two independent experiments. Statistically significant differences were calculated with one-way ANOVA and Dunnett post hoc tests. **** *p* ≤ 0.0001, ** *p* ≤ 0.01, * *p* ≤ 0.05, and ns *p* > 0.05. Figure is created by Valenzuela et al.

Although similar amounts of mouse and human SLFN14 were detected by immunoblot in cells expressing wild-type or codon-optimized Gag (Figure 6A), SLFN14 proteins decreased wild-type Gag expression by over 500 folds as compared to control cells, whereas codon-optimized Gag expression was diminished only ~2 folds (Figure 6B). Luciferase levels were also diminished by SLFN14 proteins; in cells co-expressing wild-type Gag, luciferase dropped by 17 folds and in cells co-expressing codon-optimized Gag, luciferase was diminished by ~2 folds (Figure 6B). The fact that the expression of the same luciferase transcript was ~8 folds more affected in cells co-expressing non-optimized (wild-type) than optimized Gag is intriguing. This could be due to differences in transfection efficiency, although this possibility seems to be unlikely because equal levels of SLFN14 were observed in cells expressing Gag, wild-type and codon-optimized (Figure 6A). Nevertheless, after normalization for the effect of SLFN14 proteins on luciferase (HIV-1 p24 fold reduction/Luciferase fold reduction), SLFN14 proteins still impaired expression of Gag wild type by ~36 folds, but Gag codon-optimized expression was not affected (0.9 folds). 

To better understand the molecular mechanism of SLFN14, we evaluated the effect of SLFN14 on the stability of nucleic acids extracted from the cells reported in Figure 6A,B. Importantly, purified total RNA exhibited similar RNA Integrity Number equivalent (RIN^e^) (Figure 6C(I)), indicating a comparable high quality [68]. This total RNA was used to calculate ribosomal RNA degradation with the Agilent TapeStation Controller Software 4.1 following criteria previously reported [42,57]. As compared to the corresponding control cells, cells transfected with SLFN14 showed a 1.7- or 1.1-fold increase in ribosome degradation when co-expressed with wild-type or codon-optimized Gag, respectively (Figure 6C(II)). These findings suggest that ribosome degradation could mediate the differential effects of SLFN14 on the expression of wild-type and codon-optimized Gag.

We also measured in these total RNA samples the levels of Gag mRNA using a set of primers that bind to the same region in Gag, wild-type and codon-optimized, and values were normalized to GAPDH mRNA levels in the same samples. SLFN14 diminished by 2- and 3-fold the mRNA levels in cells co-expressing Gag, wild-type and codon-optimized, respectively (Figure 6C(III)). The similar effect of SLFN14 on the stability of wild-type and codon-optimized Gag mRNA cannot explain the differential sensitivity of Gag wild-type and codon-optimized expression to SLFN14, but could explain the inhibitory effect of SLFN14 on the expression of codon-optimized Gag (Figure 6B).

We also determined the effect of SLFN14 on the stability of Gag wild-type plasmid in cells reported in Figure 6 by quantitative PCR. A similar Gag DNA cycle threshold (Ct) was observed in control cells (11.1 ± 0.1) and in SLFN14 cells (11.6 ± 0.2) (Figure 6C(IV)), indicating that SLFN14 does not trigger Gag wild-type plasmid degradation. These results also demonstrate a similar transfection efficiency in these cells.

### 3.7. Subcellular Distribution of SLFN14

**Figure 7:**
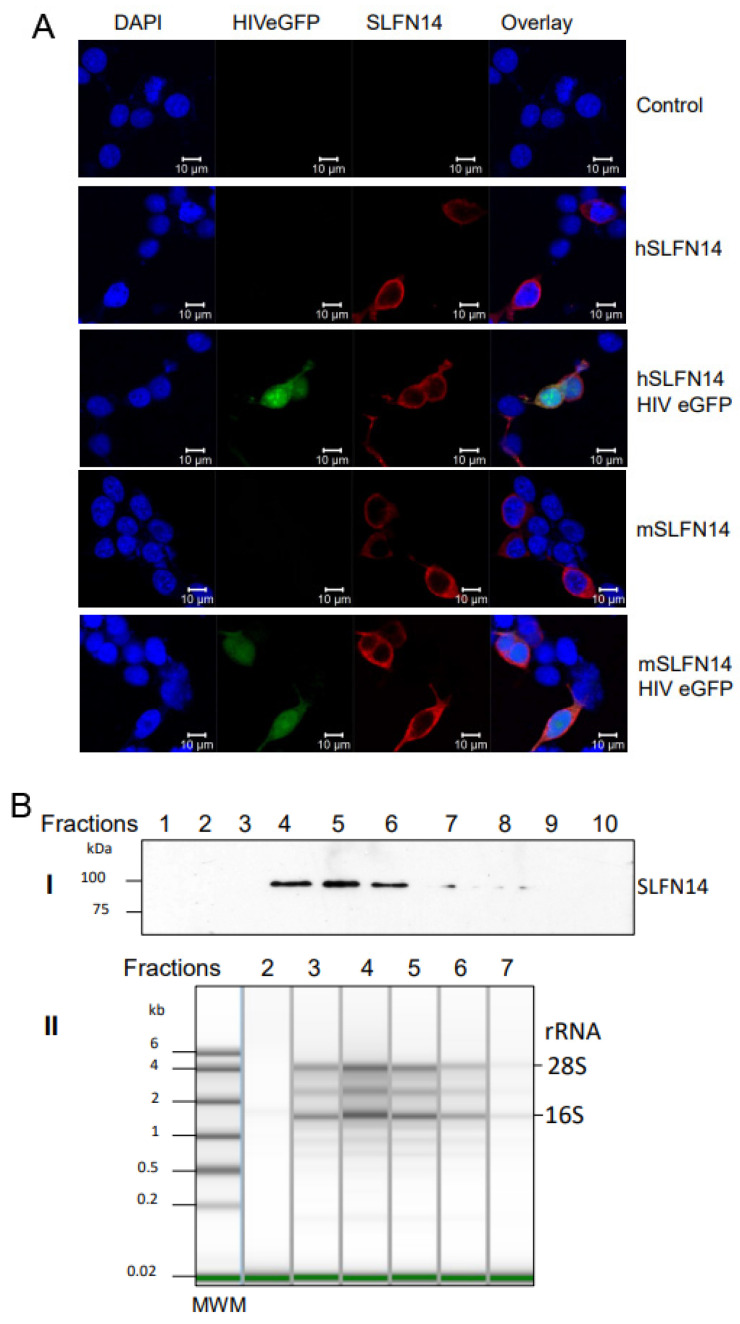
**Ribosome association of SLFN14**. (**A**) Subcellular distribution of SLFN14. SLFN14 was detected with an anti-FLAG antibody (red fluorescence) in HEK293T co-transfected with plasmids expressing mouse (m) and human (h) SLFN14 or an empty plasmid (control cells), and HIVeGFP expression plasmid or not. Nuclei were stained with DAPI. Data in the figure correspond to more than 100 cells in different fields of one experiment and are representative of three independent experiments. (**B**) Co-sedimentation of SLFN14 with ribosomes. (**I**) Immunoblotting detection of SLFN14 with an anti-FLAG antibody in sucrose gradient fractions obtained from HEK293T cotransfected with plasmids expressing human SLFN14 and HIV-1 Gag wild type. Only results obtained with fractions from the top half of the gradient are shown. (**II**) The presence of ribosomes in selected fractions was determined by RNA gel electrophoresis. Data in the figure correspond to one experiment and are representative of five independent experiments. Figure is created by Valenzuela et al.

We also determined the subcellular distribution of human and mouse SLFN14 in cells co-expressing or not HIV-1 proteins. HEK293T cells were co-transfected with a plasmid encoding a single-round infection HIV-1 expressing eGFP (HIVeGFP) [56] and either an empty plasmid or plasmids expressing mouse or human SLFN14, and SLFN14 was determined by immunostaining and confocal microscopy analysis. SLFN14 proteins were distributed exclusively to the cell cytoplasm (Figure 7A), and their localization did not change in cells co-expressing HIV-1 proteins (eGFP+ cells). 

Furthermore, we verified the association of SLFN14 with ribosomes by sedimentation analysis in a sucrose density gradient, as previously described [41,42]. Cell lysates from HEK293T co-transfected with a plasmid expressing HIV-1 wild-type Gag and either an empty plasmid or a human SLFN14 expression plasmid were resolved in a sucrose density gradient, and SLFN14 detected by immunoblot in the gradient fractions. SLFN14 was detected only in the middle region of the top quarter of the gradient (Figure 7B(I)). Gel electrophoresis analysis of RNA isolated from the SLFN14-containing fractions and the corresponding flanking fractions lacking this protein indicated the presence of SLFN14 in ribosomal RNA-enriched fractions (Figure 7B). Therefore, SLFN14 co-sedimented with ribosomal fractions, in concordance with previous reports [41,42].

## 4. Discussion

The SLFN family is widely distributed in mammals [69,70]. In mice and humans, different genes compose this family, and they are classified according to their protein length in three groups. Human SLFN group III proteins are implicated in translational control, and these functions mediate their antiviral activities [31,32,38]. SLFN11, SLFN12, and SLFN13 bind and degrade tRNAs [31,37,38,39], whereas SLFN5 binds tRNA without causing their degradation [40], and, in vitro, SLFN14, a ribosome-associated protein, degrades rRNA, tRNA, and mRNA [42]. Similarly to SLFN5, the group II protein SLFN2 binds tRNA, protecting them from stress-induced, angiogenin-mediated degradation [27]. Our findings further expand the role of this family in translational control by demonstrating in cells for the first time that SLFN14 impairs expression of transcripts enriched in rare codons at a post-transcriptional step, likely at translation. As evidenced for SLFN11 and SLFN13, this molecular function mediates the anti-HIV-1 activity of SLFN14, reported here for the first time. Similar to SLFN14, SLFN5 inhibits in a codon-usage dependent manner HIV-1 expression at a post-transcriptional step, an activity that requires its nuclease function [71].

Importantly, the effect of SLFN14 on HIV-1 protein expression was observed in several cell types, including primary cells implicated in HIV-1 infection in vivo (CD4+ T cells and monocytes) and in SUP-T1 and HEK293T cells; and on HIV-1 transcripts generated by plasmid transfection or by viral infection.

Mechanistically, our data indicate that the anti-HIV-1 activity of SLFN14 requires its endoribonuclease activity and is associated with the degradation of ribosomal RNA and Gag mRNA. Although, both Gag wild-type and codon-optimized transcripts are similarly degraded by SLFN14, ribosomal degradation occurs in SLFN14 cells co-expressing wild-type but no codon-optimized Gag. These data suggest that SLFN14 triggers degradation of ribosomes engaged in translation of codon-biased transcripts. The Gag wild-type-selective SLFN14-mediated ribosomal degradation could explain why SLFN14 impaired luciferase expression more severe in cells co-expressing wild-type than codon-optimized Gag.

SLFN14 drastically reduced the protein levels of wild-type HIV-1 Gag and firefly luciferase that exhibit a low CAI of 0.56 and 0.71, respectively, but did not affect the expression of proteins encoded by transcripts enriched in codons frequently used in human cells, such as codon-optimized HIV-1 Gag (CAI 0.99), HIV-1 Tat (CAI 0.761), CFP (CAI 0.96), mCherry (CAI 0.976), eGFP (CAI 0.962), and CD4 (CAI 0.82). Furthermore, SLFN14 more drastically impaired expression of wild-type Gag than luciferase, correlating with the lower CAI of Gag. Therefore, it is possible that SLFN14 inhibits gene expression in a rare codon-dependent manner. This would provide a mechanistic explanation to the previously reported inhibitory effect of SLFN14 on the expression of the varicella zoster virus proteins immediately early 62 and glycoprotein E [44,45] and the influenza virus nucleoprotein [45]. We have noticed that immediate-early 62, glycoprotein E, and nucleoprotein exhibit low CAIs of 0.72, 0.7, and 0.753, respectively.

Then, we postulate that, in response to translation elongation constraints experienced during the translation of codon biased mRNAs, the ribosomal-resident protein SLFN14 triggers degradation of ribosomes implicated in the translation of these messengers. We also predict that SLFN14 could also act synergistically with SLFN11, and potentially SLFN12 and SLFN13, to modulate the expression of transcripts with bias towards rare codons. In this functional crosstalk, SLFN11 and/or SLFN13 would decrease tRNA abundance globally, increasing the translation elongation constraints experienced by rare codon-enriched transcripts, making these mRNAs more susceptible to SLFN14 restriction.

Diverse cellular proteins are encoded by transcripts exhibiting codon usage bias [72]; therefore, if SLFN14 represses the expression of transcripts of this type, tight control of the SLFN14 activity or expression seems to be important. Our data indicate that HIV-1 permissive cells lack a catalytic active SLFN14 protein. SLFN14 activity is regulated in these cells by limiting the expression of the protein (i.e., HEK293T and uncultured PBMCs) or apparently by producing an N-terminal truncated form, as observed in cultured primary PBMCs, CD4+ T lymphocytes and monocytes, as well as in immortal CD4+ T cells. The proposed N-terminal truncated form is predicted to lack a region of the protein that contains the catalytic residue D249; therefore, to be non-catalytic. The truncation mechanism is unclear; RT-PCR analysis of MOLT-3 cells that express the truncated SLFN14 indicated the presence of the first nucleotides of exon 3, which encodes the potentially missing residues. In addition, the GeneBank-reported exon/intron organization does not support an alternative splicing mechanism to generate the shorter SLFN14. Therefore, the N-terminal truncated form most likely is generated by alternative initiation of translation or by post-translational processing. Unfortunately, no anti-SLFN14 antibodies, other than the antibodies we used in this research, are commercially available to further verify the hypothesis of the N-terminus truncation.

In summary, our findings indicate a novel anti-HIV-1 role of the ribosome-associated endoribonuclease SLFN14, plausibly by regulating transcript expression in a rare codon-dependent manner.

## Data Availability

Primary data is available upon request to Manuel Llano.

## Abbreviations

Schlafen (SLFN), codon adaptation index (CAI), Mean Fluorescence Intensity (MFI), RNA integrity numbers equivalent (RIN^e^), PCR cycle threshold (Ct), and horseradish peroxidase (HRP).
